# Functional status, physical activity, pain, and multimorbidity among hospitalized adults aged 65 years and older: a cross-sectional study

**DOI:** 10.3389/fpubh.2026.1781099

**Published:** 2026-05-28

**Authors:** Bożena Majchrowicz, Krystyna Kowalczuk, Robert Rak, Katarzyna Tomaszewska

**Affiliations:** 1 Collegium Medicum, Institute of Nursing, University of Rzeszów, Rzeszów, Poland; 2 Department of Integrated Medical Care, Medical University of Białystok, Białystok, Poland; 3 Collegium Masoviense, Higher School of Health Sciences, Żyrardów, Poland; 4 Department of Nursing, Institute of Health Protection, The Bronisław Markiewicz Academy of Applied Sciences, Jarosław, Poland

**Keywords:** older adults people, functional independence, multimorbidity, pain, physical activity

## Abstract

**Introduction:**

The assessment of functional performance in older adults is a key element of geriatric care and forms the basis for planning therapeutic, rehabilitative, and social interventions. Population ageing is associated with an increased prevalence of chronic diseases, reduced independence, and a higher risk of depression, all of which affect the ability of older people to function in everyday life. The aim of the study was to evaluate functional fitness and the level of physical activity in individuals aged 65+ hospitalized on non-surgical medical wards.

**Methods:**

A cross-sectional study was conducted in 2025 among 214 patients aged 65 years and older who were hospitalized in internal medicine wards. The VAS, ADL, IADL, and IPAQ scales were used. Statistical analyses included descriptive statistics, the Mann–Whitney U test, the Kruskal–Wallis test, the *χ*^2^ test, and Spearman’s rank correlation coefficient (*p* < 0.05).

**Results:**

The mean ADL score was 4.39 ± 1.61 points, while the mean IADL score was 16.19 ± 5.47 points. Most participants reported a low level of physical activity, and walking was the predominant form of activity (Me = 495 MET·min/week). Higher levels of physical activity were significantly associated with better performance in ADL and IADL (*R* = 0.812–0.887; *p* < 0.0001). A greater number of comorbidities and higher pain intensity correlated with lower physical activity (*R* = −0.215 to −0.609; *p* < 0.05) and longer sitting time (*R* = 0.500–0.639; *p* < 0.0001).

**Conclusion:**

The findings highlight the need for regular geriatric assessment, effective pain management, and interventions aimed at supporting physical activity among older adults.

## 1 Introduction

Population ageing is one of the most important demographic processes of the 21st century. The growing number of older adults is observed both in Poland and worldwide, posing a major challenge for health care and social welfare systems. As highlighted in Polish and international demographic reports, the proportion of older people in the population structure is increasing dynamically, and projections indicate a further acceleration of this trend in the coming decades (1–4). The ageing process is associated with increasing health burdens and a higher prevalence of chronic diseases such as arterial hypertension, osteoarthritis, chronic obstructive pulmonary disease, cardiovascular diseases and diabetes. Multimorbidity is one of the key challenges in geriatric medicine and has a significant impact on the level of independence and quality of life of older adults. Studies emphasize that the coexistence of chronic diseases increases the risk of mobility impairment and limitations in everyday functioning (5–9).

Functional fitness, understood as the ability to perform both basic activities of daily living and more complex instrumental activities, is one of the most important indicators of the health status of older people. A decline in the instrumental activities usually appears earlier than difficulties in basic activities, and its occurrence is strongly related to the presence of chronic diseases, age, and the level of physical activity (10–15).

Physical activity plays a crucial role in maintaining independence and good health in older age. Numerous studies indicate that regular physical activity reduces the risk of falls, improves balance, lowers the risk of cardiovascular diseases, supports mental health, and promotes the maintenance of cognitive functions (16, 17). Insufficient physical activity, on the other hand, is associated with an increased risk of functional decline, development of disability, intensification of pain, and prolonged sitting time, which constitutes a risk factor for further functional deterioration (18, 19).

In the hospitalized population, the risk of functional decline is even higher due to immobilization, the course of disease, and limited opportunities to engage in physical activity. For this reason, the assessment of functional status, degree of independence and pain intensity becomes particularly important. The tools used allow for an objective evaluation of the ability to perform activities of daily living, to determine the level of physical activity, and the assessment of pain intensity, which is a frequent and significant problem among older adults (13–16). Taking into account the growing number of older people, their specific health needs, and the important role of physical activity and comorbidities in shaping the level of functional status, a study was undertaken to comprehensively assess the functioning of patients aged 65+ hospitalized on non-surgical medical wards.

The international literature has repeatedly emphasized the association between the level of physical activity and functional capacity in older adults (20). Studies conducted in Portugal indicate that higher activity supports the maintenance of independence in instrumental activities of daily living (21). Similar observations have been reported in the Spanish population, where sedentary behaviors were shown to be associated with a deterioration in older adults’ self-sufficiency (22). Analyses from Asian countries also confirm that regular physical activity constitutes an important protective factor for maintaining functional abilities in older age (23). In turn, Irish studies indicate that, in community-dwelling populations, the proportion of individuals with ADL and IADL limitations is relatively low; however, it increases with the severity of chronic diseases (24). This is also supported by findings reported by other authors (25).

Despite numerous studies on the relationship between physical activity and functional capacity in older adults, simultaneous analyses of basic and instrumental activities of daily living, physical activity level, multimorbidity, and pain in a hospitalized patient population have rarely been conducted. Therefore, the aim of this study was to perform a multidimensional assessment of the functional performance of patients aged 65 years and older, including an analysis of independence in basic and instrumental activities of daily living, an evaluation of physical activity, and the impact of comorbidities and selected sociodemographic factors on functional performance. Based on the primary aim, the following specific objectives were formulated:

1. To analyze the relationship between the level of physical activity and functional fitness (ADL and IADL).
2. To assess the impact of comorbidities on the level of independence of the respondents.
3. To analyze sociodemographic factors differentiating the level of independence and physical activity.
4. To determine the relationships between perceived pain, multimorbidity, and the level of physical activity and sedentary behavior.

## 2 Materials and methods

### 2.1 Research design

The study was conducted in 2025 at a University Clinical Hospital in the Podkarpackie Voivodeship in Poland, covering conservative departments with internal medicine, geriatric, pulmonology, and cardiology profiles. Consecutive patients aged 65 years and older who were hospitalized in all non-surgical departments and met the inclusion criteria were eligible for participation. The study used the diagnostic survey method and a questionnaire-based technique. The research instrument was a survey questionnaire comprising questions on sociodemographic characteristics and standardized research tools: the Visual Analogue Scale (VAS), the Activities of Daily Living Scale (ADL), the Instrumental Activities of Daily Living Scale (IADL), and the International Physical Activity Questionnaire (IPAQ). Consecutive sampling was applied.

The study was exploratory in nature. The sample size was determined pragmatically based on the number of hospitalizations during the project period. At the same time, a sample size exceeding 200 participants allows for the detection of correlations of at least moderate strength (*r* ≈ 0.20–0.25) at an *α* level of 0.05 and a statistical power of 0.80. All consecutive patients who met the inclusion criteria during the study period were included, which is an acceptable approach in exploratory cross-sectional studies.

The inclusion criteria were age ≥65 years, hospitalization in a conservative department, informed consent to participate in the study, and the ability to communicate logically and provide responses. The exclusion criteria were lack of consent to participate, severe clinical condition preventing participation, and diagnosed or observed cognitive impairment that could hinder reliable completion of the questionnaires. Assessment of cognitive impairment was based on medical records, the researcher’s observation, and the patient’s ability to communicate logically and provide answers during the study. No formal screening tool, such as the MMSE or MoCA, was used, which should be considered a limitation of the study.

The sample included all available patients who met the inclusion criteria during the study period. Assistance with reading questions and marking responses was provided by members of the research team after prior instruction on neutral communication. Those providing assistance did not interpret the questions or suggest answers. The following sociodemographic variables were analyzed: age, gender, place of residence, level of education, and marital status. The study was conducted from March to August 2025.

### 2.2 Research tools

#### 2.2.1 Visual analogue scale (VAS)

To assess pain intensity, the Visual Analogue Scale (VAS) was used, which is one of the most commonly applied tools for measuring the subjective perception of pain in adults. The scale takes the form of a 10-centimetre horizontal line, whose endpoints represent: 0 – no pain and 10 – the worst pain imaginable. The respondent marks on the line the point corresponding to their current pain intensity, and the result is read in millimeters or centimeters as a numerical value. The VAS scale is characterized by high sensitivity, ease of use, and good reliability and validity, as confirmed in numerous clinical studies (13). Pain intensity experienced during daily activities and physical activity over the course of the study was assessed using the VAS.

#### 2.2.2 Activities of daily living scale (ADL)

ADL (Activities of Daily Living) scale according to Katz is a standardized tool used to assess independence in performing basic activities of daily living. It includes six categories: bathing, dressing, toileting, transferring, continence, and feeding. Each activity is assessed as being performed independently or with assistance, which makes it possible to obtain a score ranging from 0 to 6 points: 5–6 points indicated full functional independence, 3–4 points indicated moderate disability, and 0–2 points indicated severe disability. A higher score indicates a greater level of functional independence. Owing to its simplicity and good clinical utility, the scale is widely used in geriatrics, rehabilitation, and in the assessment of care needs (14).

#### 2.2.3 Instrumental activities of daily living scale (IADL)

IADL (Instrumental Activities of Daily Living) scale, developed by Lawton and Brody, is used to assess an individual’s ability to perform complex, instrumental activities of daily living that require a higher level of independence than basic ADL. The tool covers eight domains of functioning: using the telephone, shopping, food preparation, housekeeping, laundry, use of transportation, responsibility for own medication, and ability to handle finances. Each domain is rated according to the degree of independence, which allows the level of instrumental functioning to be determined. The IADL scale is widely used in geriatrics, rehabilitation, and research on functional performance, enabling the identification of early difficulties in everyday functioning. The study used the IADL scale version with a score range of 8–24 points, where 24 points indicated full independence; 19–23 points indicated mild functional limitations; 10–18 points indicated moderate functional impairment and partial dependence on assistance from others; and 0–9 points indicated substantial loss of independence and high dependence on caregivers. For IADL, Cronbach’s alpha values vary, as the scale assesses many complex domains that are only weakly interrelated. In most studies, Cronbach’s alpha ranges from 0.70 to 0.85 (15).

#### 2.2.4 International physical activity questionnaire (IPAQ)

International Physical Activity Questionnaire (IPAQ) is a standardized tool used to assess the level of physical activity in the adult population. The questionnaire is available in two versions: short and long. The assessment covers physical activity undertaken during work, transportation, household tasks, and leisure time, considering the intensity of effort (light, moderate, vigorous) and its duration over the previous 7 days. The IPAQ questionnaire referred to physical activity performed during the 7 days preceding the study, regardless of hospitalization. The results are converted into MET-minutes/week, which allows the level of physical activity to be classified as low, moderate, or high. IPAQ is widely used in epidemiological studies and in monitoring health-related behaviors in populations. IPAQ is not a psychological scale but a tool for self-reporting behaviors (16). For IPAQ, test–retest reliability is more frequently examined than Cronbach’s alpha, because the questions refer to different, independent contexts (work, transport, home, leisure), and the construction of the questionnaire does not assume high internal consistency. Nevertheless, some studies report alpha values approximately as follows: IPAQ-short: *α* ≈ 0.65–0.75; IPAQ-long: *α* ≈ 0.70–0.80.

### 2.3 Participants

A total of 238 patients were assessed, of whom 214 met the eligibility criteria and were included in the analysis. The remaining patients were excluded due to lack of consent, severe clinical condition, or difficulties in logical communication. The study was conducted among 214 patients over 65 years of age hospitalized in the University Clinical Hospital in the Subcarpathian Voivodeship, in selected non-surgical medical wards. The paper version of the questionnaire was distributed to individual patients, who were given brief instructions and informed about the purpose of the study. Some patients completed the questionnaire independently, while others requested assistance with reading the questions and recording their answers. Participation in the study was voluntary, and the data were used solely for the purposes of the present work.

### 2.4 Statistical analysis

Data analysis was carried out using the Statistica 13.3 software package. Descriptive statistics and appropriate statistical tests were applied: the chi-square test to compare qualitative variables; the Mann–Whitney U test and the Kruskal–Wallis test for between-group comparisons; and Spearman’s rank correlation coefficient to assess relationships between continuous variables. The level of statistical significance was set at *p* < 0.05. The analyses were exploratory in nature; therefore, the results, particularly those of borderline statistical significance, should be interpreted with caution.

### 2.5 Ethical procedure

Participation in the study was voluntary and anonymous. The study was conducted in accordance with the ethical standards laid down in the Declaration of Helsinki (64th WMA General Assembly, Fortaleza, Brazil, October 2013) and with Polish legal regulations. Approval to conduct the study was obtained from the Bioethics Committee of the State Academy of Applied Sciences in Przemyśl (KBPANS 15/2024).

## 3 Results

### 3.1 Characteristics of the study group

In the study population, the largest group comprised individuals aged 71–80 years (43.9%). Participants aged 65–70 years accounted for 29.9%, while those older than 80 years constituted 26.2%. Women represented 57.9% of the sample and men 42.1%. A total of 56.1% of respondents lived in rural areas, and 43.9% in urban areas. The most frequently reported educational level was vocational education (43.9%), followed by primary education (28.0%). A master’s degree was reported by 16.8% of participants and a bachelor’s degree by 11.2%. Widows and widowers constituted 42.1% of the sample. Divorced participants and those who were married each accounted for 22.4%, while 13.1% were single.

Regarding comorbidities, the most common conditions were hypertension (55.1%), diabetes (42.1%), osteoporosis (32.7%), thyroid diseases (29.9%), and COPD or asthma (26.2%). Osteoarthritis and heart failure each affected 25.2% of respondents. Malignancies were reported by 16.8%, a history of stroke by 14.0%, renal failure by 11.2%, and a history of myocardial infarction by 7.5%.

As for reasons for limited activity, 38.3% of participants indicated health-related difficulties as the main reason for being inactive. Lack of willingness was reported by 19.6%, and lack of time by 13.1%. Regular physical activity was undertaken by 29.0% of respondents. Walking was chosen by 74.8% of participants. A lack of sports-related activity was reported by 40.2%.

### 3.2 VAS scale results

The mean intensity of pain experienced by older adults during physical activity or while performing everyday tasks was 3.84 points (SD = 1.55), and the median was 4 points on a 0–10 scale. The most frequently reported pain level was 5 points, as declared by 25.2% of respondents.

### 3.3 ADL scale results

The mean score obtained by older adults on the 0–6-point scale was 4.39 points (SD = 1.61; Me = 5). Functionally independent individuals in basic activities accounted for 67.3% of the sample, 20.6% were moderately dependent, and 12.1% were severely dependent.

### 3.4 IADL scale results

The mean score obtained on the IADL scale was 16.19 points (SD = 5.47; Me = 15), with a possible range of 8–24 points. Full independence in complex activities was observed in 41.1% of respondents, 39.3% were not independent, and 19.6% of participants over 65 years of age were partially independent.

### 3.5 Impact of comorbidities on the level of independence in individuals over 65 years of age

Information on comorbidities was obtained from patients’ self-reports and medical records. Multimorbidity was analyzed as a simple count of coexisting conditions, without weighting for disease severity. A moderate or severe degree of disability in basic activities (ADL) was significantly more common among patients with osteoarthritis (*p* = 0.0034), osteoporosis (*p* = 0.0148), cancer (*p* = 0.0235), thyroid diseases (*p* = 0.0003), and COPD or asthma (*p* = 0.0232). By contrast, dependence in this area was less frequently observed among individuals reporting other, unspecified conditions (*p* = 0.0399). For the remaining diseases analyzed, no statistically significant associations were found (*p* > 0.05).

In the domain of complex activities (IADL), a lack of independence was significantly more frequent among individuals with heart failure (*p* = 0.0180), those with a history of myocardial infarction (*p* = 0.0069) or stroke (*p* = 0.0088), as well as those with osteoarthritis (*p* = 0.0042) and those with COPD or asthma (*p* = 0.0001). For other conditions, such as arterial hypertension, osteoporosis, cancer, diabetes, or renal failure, no statistically significant relationships were found between health status and the level of independence in complex activities (*p* > 0.05).

### 3.6 IPAQ scale results

In the analysis of physical activity, the median vigorous-intensity activity was 0 MET·min/week (Q1 = 0; Q3 = 120), while the median moderate-intensity activity was 200 MET·min/week (Q1 = 0; Q3 = 560). For walking, the median was 495 MET·min/week (Q1 = 149; Q3 = 891). Total physical activity reached a median of 655 MET·min/week (Q1 = 149; Q3 = 1,563). The mean daily sitting time was 457.10 ± 165.42 min. All analyzed variables showed statistically significant deviations from a normal distribution (Table 1).

**Table 1:** Physical activity in individuals aged 65 years and older (IPAQ).

Variables	M	SD	Min	Max	Q1	Me	Q3	*p*
Vigorous physical activity (MET·min/week)	92.71	160.72	0	480	0	0	120	D = 0.419; *p* < 0.0001
Moderate physical activity (MET·min/week)	295.70	292.38	0	900	0	200	560	D = 0.183; *p* < 0.0001
Physical activity related to walking (MET·min/week)	545.43	419.30	0	1,386	149	495	891	D = 0.108; *p* = 0.0035
Total physical activity (MET·min/week)	933.84	817.05	0	2,706	149	655	1,563	D = 0.171; *p* < 0.0001
Daily sitting time (min)	457.10	165.42	180	780	300	420	600	D = 0.170; *p* < 0.0001

ADL, activities of daily living; IADL, instrumental activities of daily living; IPAQ, International Physical Activity Questionnaire; MET, metabolic equivalent; M, mean; SD, standard deviation; Min, minimum; Max, maximum; Q1, first (lower) quartile; Me, median; Q3, third (upper) quartile; D, Kolmogorov–Smirnov statistic; *p*, level of statistical significance.

Statistically significant differences between the groups were found for vigorous-intensity physical activity (*p* < 0.0001), moderate-intensity physical activity (*p* < 0.0001), walking-related activity (*p* < 0.0001), and the total physical activity index (*p* < 0.0001). Significant associations were also observed between the level of functioning in basic activities of daily living and the analyzed physical activity parameters. In addition, significant between-group differences were noted in daily sitting time (*p* < 0.0001) (Table 2).

**Table 2:** Physical activity in individuals aged 65 years and older (IPAQ) and functional performance (ADL).

Variable	Degree of independence	Activities of Daily Living Scale (ADL)							*p*
		M	SD	Min	Max	Q1	Me	Q3	
Vigorous physical activity (MET·min/week)	Person with a severe or moderate disability	0.00	0.00	0	0	0	0	0	*Z* = −4.596; *p* < 0.0001
	Independent in ADL	137.78	179.63	0	480	0	0	320	
Moderate physical activity (MET·min/week)	Person with a severe or moderate disability	36.57	75.38	0	240	0	0	0	*Z* = −7.608; *p* < 0.0001
	Independent in ADL	421.67	275.16	60	900	160	360	710	
Physical activity related to walking (MET·min/week)	Person with a severe or moderate disability	164.53	166.62	0	495	33	99	297	*Z* = −7.016; *p* < 0.0001
	Independent in ADL	730.58	378.03	50	1,386	413	718	990	
Total physical activity (MET·min/week)	Person with a severe or moderate disability	201.10	228.62	0	655	33	99	347	*Z* = −7.427; *p* < 0.0001
	Independent in ADL	1290.03	760.46	110	2,706	556	1,321	1851	
Daily sitting time (min)	Person with a severe or moderate disability	604.57	171.01	290	780	360	660	720	*Z* = −5.482; *p* < 0.0001
	Independent in ADL	385.42	104.49	180	600	300	360	480	

ADL, activities of daily living; MET, metabolic equivalent; M, mean; SD, standard deviation; Q1, first (lower) quartile; Me, median; Q3, third (upper) quartile; *Z*, Mann–Whitney test statistic; *R*, rank correlation coefficient; *p*, level of statistical significance.

Additionally, Spearman’s rank correlation analysis was performed between ADL scores and physical activity parameters. Significant positive associations were found between the level of physical activity and functional status assessed using the ADL scale (*R* = 0.543–0.856; *p* < 0.0001), as well as a significant negative association between ADL scores and sitting time (*R* = −0.789; *p* < 0.0001).

Statistically significant differences between the groups were observed for all analyzed forms of physical activity as well as for sitting time (*p* < 0.0001). Significant associations were also found between the level of independence in IADL and the analyzed physical activity parameters and sitting time (*p* < 0.0001). Detailed values are presented in Table 3.

**Table 3:** Physical activity of individuals aged 65 years and older (IPAQ) and functional performance in instrumental activities (IADL).

Degree of independence		Instrumental Activities of Daily Living Scale (IADL)							*p*
		M	SD	Min	Max	Q1	Me	Q3	
Vigorous physical activity (MET·min/week)	Lack of independence	0.00	0.00	0	0	0	0	0	H = 41.056; *p* < 0.0001
	Partial Independence	57.14	128.73	0	400	0	0	0	
	Full independence	198.18	188.60	0	480	0	140	340	
Moderate physical activity (MET·min/week)	Lack of independence	62.86	92.72	0	240	0	0	160	H = 70.183; *p* < 0.0001
	Partial independence	191.43	125.47	60	420	80	160	200	
	Full independence	567.73	247.45	60	900	360	640	760	
Physical activity related to walking (MET·min/week)	Lack of independence	202.71	185.47	0	528	99	99	347	H = 56.042; *p* < 0.0001
	Partial independence	524.07	166.72	297	743	396	495	743	
	Full independence	882.75	395.35	50	1,386	660	941	1,155	
Total physical activity (MET·min/week)	Lack of independence	265.57	267.14	0	768	99	99	408	H = 62.945; *p* < 0.0001
	Partial independence	772.64	362.22	493	1,563	497	695	803	
	Full independence	1648.66	738.13	110	2,706	1,280	1731	2,311	
Daily sitting time (min)	Lack of independence	590.71	153.76	360	780	420	630	720	H = 53.069; *p* < 0.0001
	Partial independence	447.62	105.59	290	600	360	420	540	
	Full independence	334.09	82.25	180	480	300	300	360	

IADL, instrumental activities of daily living; MET, metabolic equivalent; M, mean; SD, standard deviation; Q1, first (lower) quartile; Me, median; Q3, third (upper) quartile; *H*, Kruskal–Wallis test statistic; *R*, rank correlation coefficient; *p*, level of statistical significance.

Spearman’s rank correlation analysis showed significant positive associations between the level of physical activity and functional status assessed using the IADL scale (*R* = 0.634–0.887; *p* < 0.0001), as well as a significant negative association between IADL scores and sitting time (*R* = −0.828; *p* < 0.0001). Significant correlations were also identified between the number of comorbidities, pain intensity, and both physical activity level and sitting time (Table 4).

**Table 4:** Physical activity of individuals aged 65 years and older (IPAQ) in relation to multimorbidity and pain intensity.

Variables		Multimorbidity	Pain experienced in relation to physical activity or the performance of usual activities among individuals aged 65 years and older
Vigorous physical activity (MET·min/week)	*R*	−0.176	−0.510
	*p*	0.0698	<0.0001
Moderate physical activity (MET·min/week)	*R*	−0.317	−0.609
	*p*	0.0009	<0.0001
Physical activity related to walking (MET·min/week)	*R*	−0.156	−0.525
	*p*	0.1094	<0.0001
Total physical activity (MET·min/week)	*R*	−0.215	−0.580
	*p*	0.0262	<0.0001
Daily sitting time (min)	*R*	0.500	0.639
	*p*	<0.0001	<0.0001

MET, metabolic equivalent; *R*, Spearman’s rank correlation coefficient; *p*, level of statistical significance.

Spearman’s rank correlation coefficients showed that a higher number of comorbidities was significantly associated with lower levels of moderate and total physical activity, as well as with longer sitting time. However, no significant associations were found between multimorbidity and vigorous physical activity or walking-related activity. Greater pain intensity was associated with lower levels of all analyzed forms of physical activity and with longer sitting time.

### 3.7 Sociodemographic factors and functional status

Significant differences between age groups were found for ADL (*p* = 0.0112) and IADL (*p* < 0.0001). Lower functional status scores were observed particularly among participants aged over 80 years. With increasing age, the declared level of physical activity also decreased, while sitting time increased.

Gender did not significantly differentiate the level of functional status or physical activity (*p* > 0.05). However, significant associations were observed with place of residence and level of education (*p* < 0.05). Participants with higher education more often reported greater physical activity and a higher level of independence in activities of daily living. Differences related to place of residence indicated a higher level of physical activity among urban residents.

Statistically significant differences were also observed according to marital status (*p* < 0.0001). Married participants more often demonstrated a higher level of functional independence than those who were single, widowed, or divorced (see Table 5).

**Table 5:** Sociodemographic factors and the functional status of the study participants.

Sociodemographic factor	ADL *χ*^2^	*p*	IADL *χ*^2^	*p*
Age	16.471	0.0024	46.522	<0.001
Gender	31.533	<0.001	7.992	0.0919
Place of residence	27.979	<0.001	34.782	<0.001
Education	39.162	<0.001	49.437	<0.001
Marital status	42.100	<0.001	57.853	<0.001

*χ*^2^, chi-square test statistic. Values of *p* < 0.05 were considered statistically significant.

### 3.8 Multivariable analysis

Additionally, a multivariable regression analysis was performed, in which the ADL score was the dependent variable, while the independent variables included the level of physical activity, pain intensity, number of comorbidities, age, and sex. The aim of the analysis was to assess independent predictors of functional status.

In the multivariable analysis, a higher level of physical activity remained independently associated with a better ADL score, whereas greater pain intensity and a higher number of comorbidities were associated with lower functional status after adjustment for age and sex (see Table 6).

**Table 6:** Multivariable linear regression analysis for functional status assessed using the ADL scale.

Predictor	*β*	*p*
Total IPAQ score	0.48	<0.001
VAS pain score	−0.31	0.002
Multimorbidity	−0.22	0.01
Age	−0.19	0.03
Gender	NS	0.44

## 4 Discussion

The aim of the study was to conduct a multidimensional assessment of the relationships between the level of physical activity, performance in basic and complex activities of daily living, multimorbidity, pain intensity, and sociodemographic factors among patients aged 65 years and older hospitalized in medical (non-surgical) wards. The findings indicate close interrelationships between the analyzed domains.

The study showed that functionally independent individuals achieved significantly higher levels of physical activity and had shorter sitting time. This association applied to both basic and instrumental activities. These results are consistent with observations reported by other authors, who emphasize that regular physical activity supports the maintenance of independence and delays the development of disability (26–28). It should be noted that most available studies concern community-dwelling populations, whereas analyses including hospitalized patients are less common.

The clinical condition of this group, greater severity of chronic diseases, and periodic limitations in mobility may contribute to stronger associations between physical activity and functional status than those observed in the general population. The present findings complement existing knowledge by providing data on a particularly vulnerable group of hospitalized patients.

One possible explanation for the observed associations is the coexistence of functional limitations and reduced physical activity. Lower activity levels may contribute to decreased muscle strength and physical capacity, which in turn may hinder the performance of daily activities and lead to further restrictions in physical activity. Similar conclusions were presented by Sánchez-Sánchez et al. (22), who indicated the negative impact of sedentary behavior on the level of independence.

The obtained data indicate that a higher number of chronic diseases was mainly associated with lower levels of moderate and total physical activity, as well as with longer sitting time. However, no significant associations were found between multimorbidity and vigorous physical activity or walking-related activity. This relationship is widely described in the literature. Rizzuto et al. and Guido et al. showed that multimorbidity is one of the key factors contributing to loss of functional ability and increased dependence on assistance from others (29, 30). As the number of diseases increases, the frequency of pain, dyspnea, and fatigue also rises, which may lead to avoidance of physical exertion and further deterioration in functioning (31, 32).

In the present study, more severe pain symptoms were associated with lower physical activity and greater sedentary behavior. This observation is supported by the findings of Ćwirlej-Sozańska et al. (33), who identified pain as one of the most important modifiable risk factors for disability. Movement limitation due to pain can result in muscle weakness, impaired balance, and further loss of independence.

With increasing age, a clear decline in functional capacity was observed, particularly with respect to complex activities. This trend is consistent with numerous reports emphasizing that advanced age is associated with an accumulation of health deficits and a reduction in physiological reserves (17, 24, 34).

The significant role of education in the level of independence has also been confirmed in other studies. Fidecki et al. (17) indicated that higher educational attainment promotes better functioning, which may result from greater health awareness and more frequent use of healthcare services.

In studies conducted in community-dwelling populations, the proportion of individuals with ADL and IADL limitations is often lower than in hospitalized groups. Connolly et al. (24) showed that only a small proportion of older adults reported difficulties in performing activities of daily living. These differences may result from the poorer health status of patients requiring hospital treatment, a greater burden of disease, and temporary immobilization.

It may be assumed that, in hospital settings, even minor differences in functional status may translate into the ability to engage in activity. Patients with lower functional status more often require assistance from medical staff, are less likely to leave the bed, and may experience concerns related to deterioration of their health, which may further contribute to reduced physical activity.

The present findings are consistent with reports showing that regular physical activity supports the maintenance of independence in older age. Studies have demonstrated that exercising at least three times per week may be associated with a higher level of functional independence among older adults (26). Baek et al. (27) emphasized that regular resistance exercise, including progressive programs, may not only improve functional capacity and the ability to perform activities of daily living, but also positively affect mood and reduce depressive symptoms. Tornero-Quiñones et al. (28) likewise showed that physically active individuals achieved significantly better functional performance compared with those leading a less active lifestyle. These findings reinforce the results of the present study, highlighting the important role of regular physical exertion in maintaining independence in older adults.

The present results address a gap identified in the literature, as they simultaneously analyze basic and complex activities of daily living, physical activity level, pain-related symptoms, and multimorbidity in a hospitalized patient population. Such a comprehensive approach enables a better understanding of the co-occurrence of factors affecting the maintenance of independence in older age and constitutes an important complement to previous research conducted mainly in community-dwelling populations. The findings also underscore the need for systematic functional assessment of older patients. Early identification of limitations, adequate pain management, and the implementation of activation programs may help slow the process of functional decline and improve older adults’ quality of life. In clinical practice, this may entail the early introduction of rehabilitative interventions already in the first days of hospitalization. Even simple measures - such as assisted standing, short walks along the ward, or low-intensity exercises - may help limit the progression of disability.

## 5 Limitations of the study

Due to the cross-sectional nature of the study, it was not possible to determine the direction of causal relationships. The relationship between physical activity and functional status may be bidirectional. Non-probability consecutive sampling was used, which may have increased the risk of selection bias. Data on physical activity and pain were self-reported, which may be associated with recall bias and subjectivity of responses. Cognitive function, as well as environmental and social conditions that could have influenced the participants’ level of independence and physical activity, were not assessed. The study was conducted in a single hospital and among hospitalized patients, which limits the generalizability of the findings to the general older adult population. Hospitalization may have influenced the declared level of physical activity assessed using the IPAQ. An additional limitation is that multiple statistical analyses were performed without correction for multiple testing, which may increase the risk of type I error.

## 6 Implications for practice

The findings indicate the necessity of routinely using comprehensive geriatric assessment, which enables early detection of reduced functional status and identification of risk factors for loss of independence. Particularly important is the development of physical activity programmes tailored to the abilities of older adults, as regular exercise supports the maintenance of independence in both basic and instrumental activities. An essential element of practice is also effective pain management and appropriate treatment of chronic diseases that limit mobility in older adults.

Education of patients and their families regarding the benefits of physical activity and the prevention of immobility is indispensable, as is the consideration of psychosocial aspects of functioning, such as loneliness or low mood. In addition, creating environments that promote the activation of older adults – both at home and in the community – may provide a basis for planning interventions aimed at supporting functional status.

## 7 Conclusion

Physical activity was significantly associated with the level of functional performance in hospitalized older adults, whereas multimorbidity and pain intensity were linked to lower physical activity and greater sedentary behavior. These findings underscore the need for routine functional assessment and for implementing activation and pain-management interventions as part of comprehensive geriatric care.

## Data Availability

The raw data supporting the conclusions of this article will be made available by the authors, without undue reservation.
